# SERVED BY SYSTEMS: AGING WITH INTELLECTUAL AND DEVELOPMENTAL DISABILITIES AND INEQUITIES IN HEALTHCARE

**DOI:** 10.1093/geroni/igac059.3055

**Published:** 2022-12-20

**Authors:** Danielle Waldron, Emily Hartford, Jacquelin Sauer, Jeffrey Stokes

**Affiliations:** Stonehill College, Taunton, Massachusetts, United States; Stonehill College, Easton, Massachusetts, United States; Stonehill College, Easton, Massachusetts, United States; University of Massachusetts Boston, Boston, Massachusetts, United States

## Abstract

Adults and older adults with intellectual and developmental disabilities (I/DD) often experience medical complexity, accelerated aging, and shortened life expectancy. This project explores health service utilization (HSU) of adults with I/DD receiving state services in the U.S. over the adult lifespan before and after the implementation of the Affordable Care Act (ACA). Three waves of cross-sectional data (2008–2019) from the National Core Indicators-In Person Survey were analyzed using multilevel mixed effects logistic regression (n=46,284). Older adults with I/DD were more likely to receive a physical exam, flu vaccine, eye exam, hearing test, and a dental exam compared to younger individuals, although these differences were small. Compared to non-Hispanic whites, non-Hispanic blacks were less likely to receive physical exams, flu vaccines, and dental exams; non-Hispanic other were less likely to receive eye exams and dental exams; and Hispanic persons were less likely to receive eye exams, flu vaccines, and dental exams. Individuals with I/DD living in states that expanded Medicaid were at 68% greater odds of receiving a physical exam than those who did not. Our research indicates that overall adults with I/DD are not yet reaching HSU of pre-ACA times, perhaps due to the oversaturation of existing providers. National and state policies, along with individual case management each play a role in ensuring healthy aging of individuals with I/DD. A call to action to better understand and integrate these three entities may help improve the potential for healthy aging of this group.

